# The potential impact of moxidectin on onchocerciasis elimination in Africa: an economic evaluation based on the Phase II clinical trial data

**DOI:** 10.1186/s13071-015-0779-4

**Published:** 2015-03-19

**Authors:** Hugo C Turner, Martin Walker, Simon K Attah, Nicholas O Opoku, Kwablah Awadzi, Annette C Kuesel, María-Gloria Basáñez

**Affiliations:** London Centre for Neglected Tropical Disease Research, Department of Infectious Disease Epidemiology, School of Public Health, Faculty of Medicine, Imperial College London, St. Mary’s Campus, Norfolk Place, London, W2 1PG UK; Department of Infectious Disease Epidemiology, School of Public Health, Faculty of Medicine, Imperial College London, St Mary’s Campus, Norfolk Place, London, W2 1PG UK; Onchocerciasis Chemotherapy Research Centre, Hohoe, Ghana; Department of Microbiology, University of Ghana Medical School, Accra, Ghana; UNICEF/UNDP/World Bank/ WHO Special Programme for Research and Training in Tropical Diseases, World Health Organization, Geneva, Switzerland

**Keywords:** Onchocerciasis, Moxidectin, Ivermectin, Economic evaluation, Seasonality

## Abstract

**Background:**

Spurred by success in several foci, onchocerciasis control policy in Africa has shifted from morbidity control to elimination of infection. Clinical trials have demonstrated that moxidectin is substantially more efficacious than ivermectin in effecting sustained reductions in skin microfilarial load and, therefore, may accelerate progress towards elimination. We compare the potential cost-effectiveness of annual moxidectin with annual and biannual ivermectin treatment.

**Methods:**

Data from the first clinical study of moxidectin were used to parameterise the onchocerciasis transmission model EPIONCHO to investigate, for different epidemiological and programmatic scenarios in African savannah settings, the number of years and in-country costs necessary to reach the operational thresholds for cessation of treatment, comparing annual and biannual ivermectin with annual moxidectin treatment.

**Results:**

Annual moxidectin and biannual ivermectin treatment would achieve similar reductions in programme duration relative to annual ivermectin treatment. Unlike biannual ivermectin treatment, annual moxidectin treatment would not incur a considerable increase in programmatic costs and, therefore, would generate sizeable in-country cost savings (assuming the drug is donated). Furthermore, the impact of moxidectin, unlike ivermectin, was not substantively influenced by the timing of treatment relative to seasonal patterns of transmission.

**Conclusions:**

Moxidectin is a promising new drug for the control and elimination of onchocerciasis. It has high programmatic value particularly when resource limitation prevents a biannual treatment strategy, or optimal timing of treatment relative to peak transmission season is not feasible.

**Electronic supplementary material:**

The online version of this article (doi:10.1186/s13071-015-0779-4) contains supplementary material, which is available to authorized users.

## Background

The primary goal of onchocerciasis control in Africa has recently shifted from morbidity prevention to elimination of the infection where possible by 2025 [1], including elimination of onchocerciasis in selected African countries by 2020 [2]. Currently, the predominant control strategy in Africa is preventative chemotherapy by annual community-directed treatment with ivermectin (aCDTI). Merck & Co committed to donate ivermectin for as long as needed to eliminate the public health burden of onchocerciasis [3].

The global health community recognises that the reservoir of *Onchocerca volvulus* will not be eliminated in all endemic foci in Africa with aCDTI alone, and that new tools and strategies are needed [2]. In the 13 endemic foci in Latin America (population at risk approximately 0.56 million), biannual ivermectin mass treatment (complemented in certain hyperendemic areas with more frequent administration) has, or is likely to have, interrupted transmission in 11 foci [4,5]. Biannual CDTI (bCDTI) could improve the chances of achieving elimination in Africa, which has a population at risk of onchocerciasis of approximately 115 million people [6]. In some foci in Senegal 17 years of bCDTI interrupted onchocerciasis transmission [7,8]. Ghana and Uganda currently implement bCDTI in selected foci [9,10], and bCDTI was also used in several of the Special Intervention Zones after the closure of the former Onchocerciasis Control Programme in West Africa (OCP) [11].

A previous modelling study [12] indicated that although bCDTI yields only small additional health benefits rela-tive to aCDTI, it substantially reduces the number of years required to reach the provisional operational thresholds for treatment interruption followed by sur-veillance (pOTTIS) [13]. This reduction is most pronounced in areas with very high pre-control skin microfilarial prevalence, where model projections sug-gest that elimination would not be possible with over 50 years of aCDTI. Furthermore, bCDTI would reduce the difference in years to reach the pOTTIS among areas with very different pre-control endemicities [12]. This is noteworthy since CDTI should only be stopped when there is sufficiently low risk of re-introduction of the parasite from neighbouring areas.

A subsequent and recent modelling study has also found that increasing the treatment frequency of ivermectin to twice per year notably reduces the programme duration (also by about 35% in mesoendemic and hyperendemic settings) [14]. Though these reductions were found to be highly dependent on the level of maintained coverage, and could be completely nullified if coverage were to fall [14].

In Ghana, bCDTI has increased programmatic costs by 50-60% per year relative to aCDTI [15]. Consequently, even with a marked reduction in the number of years to reach the pOTTIS, model projections indicate that bCDTI in many areas will have a higher total cost than aCDTI [12]. Furthermore, bCDTI may not always be feasible, particularly where resources are scarce or access to communities is only possible during dry seasons.

Moxidectin is a highly efficacious veterinary anthelmintic [16] and a potential alternative to ivermectin for preventive chemotherapy and elimination of human onchocerciasis. In a Phase II clinical trial, moxidectin reduced skin microfilarial loads to statistically significantly lower levels and for substantially longer than ivermectin [17]. The effect through 1 year after treatment supports the hypothesis that annual community-directed treatment with moxidectin (aCDTM) has an effect on transmission comparable to that of bCDTI.

We tested this hypothesis by modelling aCDTI, bCDTI and aCDTM strategies, assessing the time and cost to reach the pOTTIS under a variety of epidemiological and programmatic conditions. These included, for the first time, the effect of the timing of treatment relative to seasonal transmission patterns. In some foci, the breeding sites of the simuliid vectors dry up and biting rates dwindle to zero, potentially decreasing the effectiveness of ivermectin treatment if it is not timed to ensure minimal skin microfilarial levels when biting rates are highest [7,8].

## Methods

### Onchocerciasis transmission model

The modelling was undertaken using EPIONCHO, a host sex- and age-structured deterministic onchocerciasis transmission model [18,19], parameterised for African savannah settings [18]. The underlying demography is that of northern Cameroon, assuming a stationary age distribution and a stable (closed) population [18].

For all modelling not aimed at assessing the impact of treatment timing relative to transmission season, perennial transmission (all year round) was assumed (Table 1). To model seasonal peaks in transmission, the biting rate of blackfly (*Simulium damnosum*) vectors was allowed to vary throughout the year (Additional file 1: Table S1 and Additional file 1: Figure S1).

**Table 1:** **Summary of factors whose impact was modelled on the duration and cost of reaching the pOTTIS**

**Parameters**	**Values**
Seasonality of transmission (see Supporting information (Additional file 1: Text S.1, and Additional file 1: Figure S1))	Perennial transmission: annual biting rate (ABR) is constant throughout the year (i.e. no seasonal changes)
	Seasonal transmission scenario 1: transmission occurs during a rainy season typically lasting approximately five months each year; based on foci in Senegal and Mali [7,8] were elimination has been reported
	Seasonal transmission scenario 2: a longer period of transmission, still peaking in the rainy season but not ceasing completely in the dry season; motivated by the entomological observations in [40]
The proportion of the total population receiving ivermectin or moxidectin at each treatment round, referred to as therapeutic coverage	60% and 80%
The proportion of the eligible population who never receive treatment, referred to as the proportion of systematic non-compliers	0.1%, 2% and 5%
The discount rate applied to the costs [23]	0%, 3% and 6%
The per treatment round cost of aCDTM relative to aCDTI	100% (i.e. the same) and 110%
The per dose (cumulative) reduction in microfilarial production of female adult worms, referred to as anti-macrofilarial action of ivermectin	1%, 7% and 30%
Provisional Operational Thresholds for Transmission Interruption followed by Surveillance (pOTTIS)	0.9%, 1.4% and 1.9% microfilarial prevalence (i.e. 1.4% ± 0.5%)

aCDTM: annual community-directed treatment with moxidectin; aCDTI: annual community-directed treatment with ivermectin; pOTTIS: provisional operational thresholds for treatment interruption followed by surveillance.

### Drug effects

**Figure 1:**
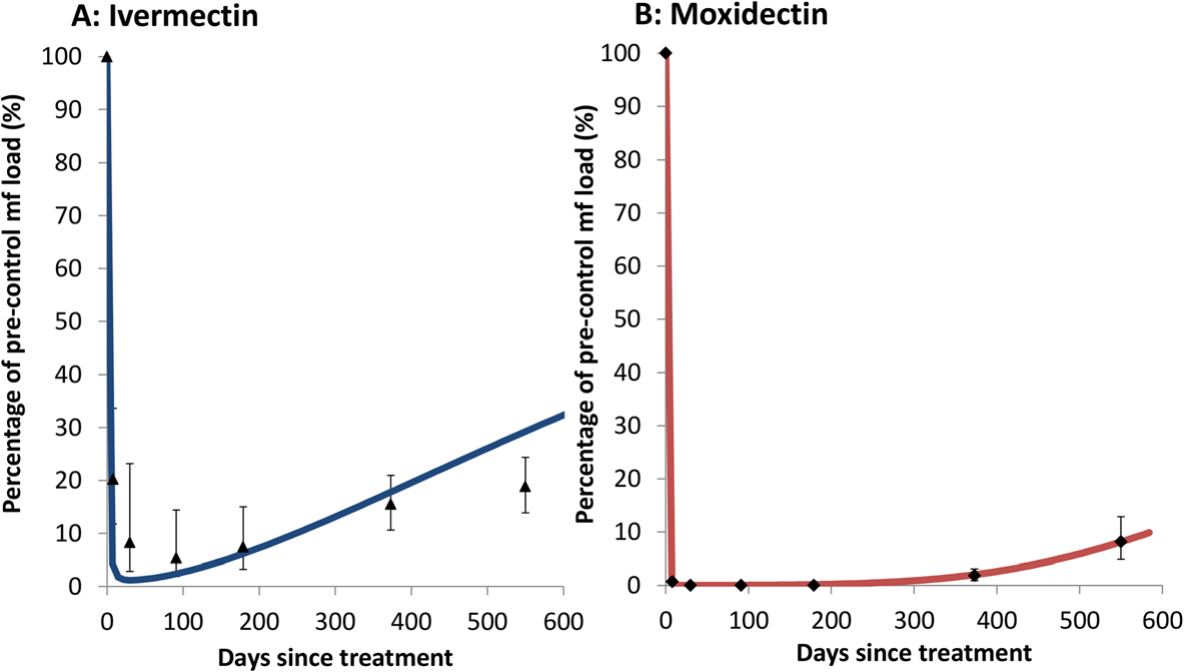
**The dynamic effect of a single dose of ivermectin (A) and moxidectin (B) on skin microfilarial load.** The data points are derived from skin microfilarial loads (the mean of four microfilarial counts [17]) collected from **(A)** the 45 control participants (who took ivermectin) and **(B)** the 38 treated participants (who took moxidectin) as part of the Phase II clinical safety trial of moxidectin for the treatment of onchocerciasis [17]. The effect of a single dose of ivermectin previously fitted to microfilarial load data collated as part of a meta-analysis [20] is shown as the solid blue line in **(A)** (note that the microfilarial dynamics induced by ivermectin are not re-estimated here and hence provide a validation of the previous parameterization). The dynamical effect precipitated by moxidectin was fitted to the trial data on microfilarial loads from treated participants using the same approach as in [20] (described in the Supporting information (Additional file 1: Text S.2)) and is shown as the solid red line in **(B)**. Error bars show the 95% confidence intervals which in some circumstances were narrower that the plotted data point and so are not discernible.

EPIONCHO incorporates the temporal dynamics of the microfilaricidal and embryostatic (temporary sterilisation of female worm) effects of ivermectin, based on previous modelling of data from clinical and community trials of ivermectin (Figure 1A) [20]. The temporal dynamics of skin microfilarial loads from the ivermectin treatment arm in the Phase II moxidectin study were within the range observed in [20]. Moxidectin treatment was assumed to exert the same types of effects on the parasite as ivermectin. Therefore, moxidectin’s effects were parameterised by fitting the functions in [20] to the percentage reduction in skin microfilarial densities from pre-treatment, measured 8 days, 1, 2, 3, 6, 12 and 18 months after a single dose of 8 mg moxidectin (91–186 μg/kg or 0.14-0.29 μmol/kg for the weight range included) in 38 adult hosts [17] (Figure 1B, Additional file 1: Table S2).

Given the uncertain, and somewhat controversial [19,21] cumulative effect of repeated ivermectin exposure of adult worms on their rate of microfilarial production (anti-macrofilarial action), modelling was conducted assuming 7% cumulative reduction per standard dose of ivermectin ( 150 μg/kg or 6, 9 or 12 mg, 0.16-0.23 μmol/kg for the weight range in the 8 mg moxidectin arm of the Phase II study) as well as extreme values of 1% and 30% [19,21] (Table 1). No data exist on the effect of multiple doses of moxidectin, so we assumed the same cumulative anti-macrofilarial effect size as for ivermectin. (Note that the embryostatic effect is assumed to be temporary, whereas the anti-macrofilarial action is assumed to be cumulative.) It was also assumed that, like ivermectin, individuals aged > = 5 years would be eligible for moxidectin treatment.

### Operational thresholds for treatment interruption followed by surveillance (pOTTIS)

The African Programme for Onchocerciasis Control (APOC) conceptual and operational framework for elimination [13] proposed provisional operational thresholds for treatment interruption followed by surveillance (pOTTIS). They assess the residual level of patent infection in the human population (skin snip-based microfilarial prevalence of <5% in all surveyed villages and <1% in 90% of surveyed villages), and fly infectivity (<0.5 infective larvae per 1,000 blackflies). The pOTTIS are not necessarily equivalent to a transmission breakpoint, a hypothetical parasite density below which the population would be unable to maintain itself [22].

As in our previous analysis of bCDTI [12], we assumed that the pOTTIS were reached when the modelled microfilarial prevalence (all ages), measured just before the next treatment round, fell below 1.4%, the weighted average of the pOTTIS prevalence thresholds. We used the microfilarial prevalence thresholds because in our simulations the entomological threshold was always reached sooner [12], making the former more conservative. Since the pOTTIS are provisional [13], we also modelled pOTTIS of 0.9% and 1.9% microfilarial prevalence (Table 1).

### In-country costs

The economic cost of aCDTI was set at US$41,534 per 100,000 individuals (overall target population) per year (2012 prices). This increased by 60% for bCDTI. These costs were estimated from data collected in Ghana [15] and are those incurred by the Ministry of Health, non-government organization (NGO) partners and volunteer community distributors. (The health care providers’ perspective was chosen because the costs to the local community for accessing treatment should be negligible.) The economic value of donated ivermectin was not included [15].

The cost of aCDTM was assumed to be either identical to that of aCDTI or 10% higher to account for potential extra costs of social mobilization and training to distribute a new drug. It was assumed that moxidectin would, like ivermectin, be donated to endemic countries.

Following WHO guidelines [23], a discount rate of 3% was applied to the costs. Discounting deflates costs incurred in the future to reflect that society prefers to delay costs rather than incur them in the present.

### Scenarios modelled

EPIONCHO was used to project the number of years of treatment required to reach the pOTTIS (programme duration) and the associated in-country costs with aCDTI, bCDTI and aCDTM over a 50-year time horizon for a range of initial endemicity levels (40%, mesoendemic; 60%, hyperendemic; 80%, highly hyperendemic pre-control microfilarial prevalence; Additional file 1: Table S3). For each endemicity level, programme duration and cost were subjected to a sensitivity analysis (Table 1). In addition, different timing of aCDTI and aCDTM treatment relative to peak transmission was modelled for two seasonal transmission scenarios (see Supporting information).

## Results

**Figure 2:**
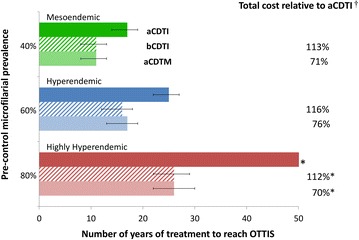
**Comparison of annual and biannual community-directed treatment with ivermectin (aCDTI, bCDTI) versus annual community-directed treatment with moxidectin (aCDTM) in areas where control has not been previously implemented.** The green, blue and red bars correspond to a pre-control endemicity level of 40%, 60%, and 80% microfilarial prevalence, respectively. The aCDTI, bCDTI and aCDTM strategies are indicated by solid, dashed and dotted bars respectively. Error bars represent the results of varying the provisional operational thresholds for treatment interruption followed by surveillance (pOTTIS) by adding or subtracting 0.5% (i.e. 0.9% or 1.9% microfilarial prevalence). Results shown assume a therapeutic coverage of 80%; a proportion of systematic non-compliers of 0.1%; perennial transmission, and a 7% per dose (cumulative) reduction in microfilarial production of female adult worms. A discount rate of 3% was applied to the costs. *pOTTIS (1.4% microfilarial prevalence) not attained within the 50-year time horizon and percentage of costs calculated based on costs of 50 years of aCDTI. † Costs do not include value of the (donated) drugs.

**Figure 3:**
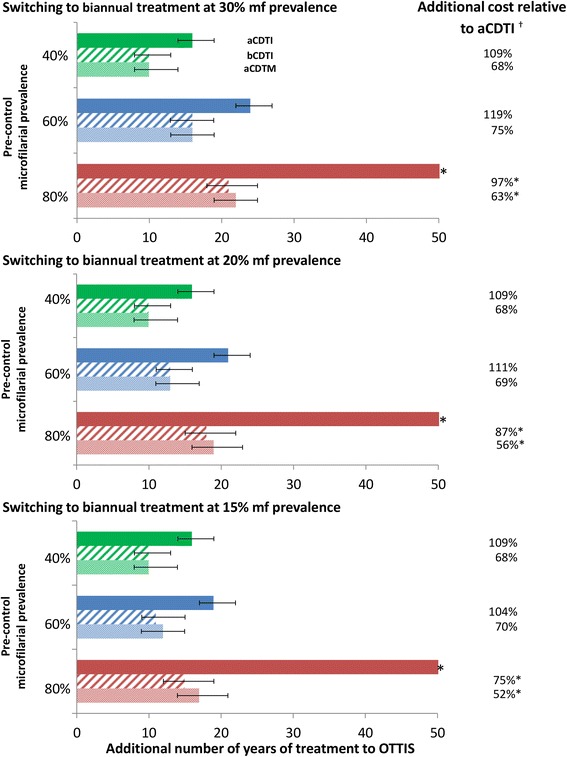
**Impact of switching to biannual community-directed treatment with ivermectin (bCDTI) or annual community-directed treatment with moxidectin (aCDTM) at different stages of an ongoing annual CDTI (aCDTI) programme.** The green, blue and red bars correspond to a pre-control endemicity level of 40%, 60%, and 80% microfilarial prevalence, respectively. The aCDTI, bCDTI and aCDTM strategies are indicated by solid, dashed and dotted bars, respectively. Error bars represent the results of varying the provisional operational thresholds for treatment interruption followed by surveillance (pOTTIS) by ± 0.5%. The number of additional years of treatment and the ratio of additional costs are considered from the point of switching to bCDTI or aCDTM (and not from the start of control). Modelling assumptions are as in the legend of Figure 2. *pOTTIS (1.4% microfilarial prevalence) not attained within the 50-year time horizon and percentage of costs calculated based on costs of 50 years of aCDTI. † Costs do not include value of the (donated) drugs.

In the Phase II clinical trial, a single dose of 8 mg moxidectin reduced pre-treatment skin microfilarial levels by 98%-100% from 8 to 365 days after treatment (Figure 1B, [17]). This higher and more prolonged efficacy compared to ivermectin (Figure 1A) resulted in shorter simulated programme durations for aCDTM than aCDTI. This was found to apply both when aCDTM is used from the outset (Table 2, Figure 2) and when a switch from aCDTI to aCDTM is made during ongoing control activities (Figure 3, Additional file 1: Table S4). The programme durations with aCDTM were comparable to those with bCDTI. For both bCDTI and aCDTM, the reductions in programme duration relative to aCDTI increased with increasing baseline prevalence of infection (Figure 2, Figure 3, Additional file 1: Table S4), i.e. the benefits of more effective strategies accrued disproportionately with increasing initial endemicity. Like bCDTI [12], aCDTM reduced the difference in programme duration between areas with different pre-control endemicities relative to aCDTI (Figure 2, Figure 3, Additional file 1: Table S4). The decrease and increase in programme duration when increasing or decreasing, respectively, the pOTTIS, from 1.4% to 1.9% or 0.9% average skin microfilarial prevalence were similar for all three strategies (as indicated by the error bars in Figures 2 and 3).

**Table 2:** **Sensitivity of the duration (time to achieve pOTTIS) and relative total cost of annual ivermectin (aCDTI), biannual ivermectin (bCDTI) and annual moxidectin (aCDTM) treatment programmes to the magnitude of the assumed anti-macrofilarial action of ivermectin and moxidectin**

**Baseline endemicity level (microfilarial prevalence)**	**1% cumulative reduction in microfilarial production by female adult worms per dose**			**7% cumulative reduction in microfilarial production by female adult worms per dose**			**30% cumulative reduction in microfilarial production by female adult worms per dose**		
	**Projected duration, in years, of treatment programme (relative cost, in percent)**			**Projected duration, in years, of treatment programme (relative cost, in percent)**			**Projected duration, in years, of treatment programme (relative cost, in percent)**		
	aCDTI	bCDTI (ǂ)	aCDTM (ǂ,†)	aCDTI	bCDTI (ǂ)	aCDTM (ǂ,†)	aCDTI	bCDTI (ǂ)	aCDTM (ǂ,†)
Mesoendemic (40%)	21	14 (118%)	12 (65%, 55%)	17	11 (113%)	11 (71%, 63%)	12	9 (126%)	10 (86%, 68%)
Hyperendemic (60%)	33	20 (115%)	18 (67%, 58%)	25	16 (116%)	17 (76%, 66%)	17	14 (138%)	15 (91%, 66%)
Highly-hyperendemic (80%)	NA	38 (140%)	30 (76%, 54%)	NA	26 (112%)	26 (70%, 63%)	38	22 (114%)	23 (74%, 65%)

^ǂ^Percentage cost relative to aCDTI. ^†^Percentage cost relative to bCDTI. NA: Operational thresholds for treatment interruption not attained within the 50-year time horizon (and percentage of costs calculated based on costs of 50 years of aCDTI). The analysis was performed with a 50-year time horizon, therapeutic coverage of 80%, 0.1% systematic non-compliers, perennial transmission, and pOTTIS of <1.4% microfilarial prevalence. Costs do not include value of the (donated) drugs. A summary of the pre-control conditions is provided in Additional file 1: Table S3.

### Impact of therapeutic coverage and compliance

**Figure 4:**
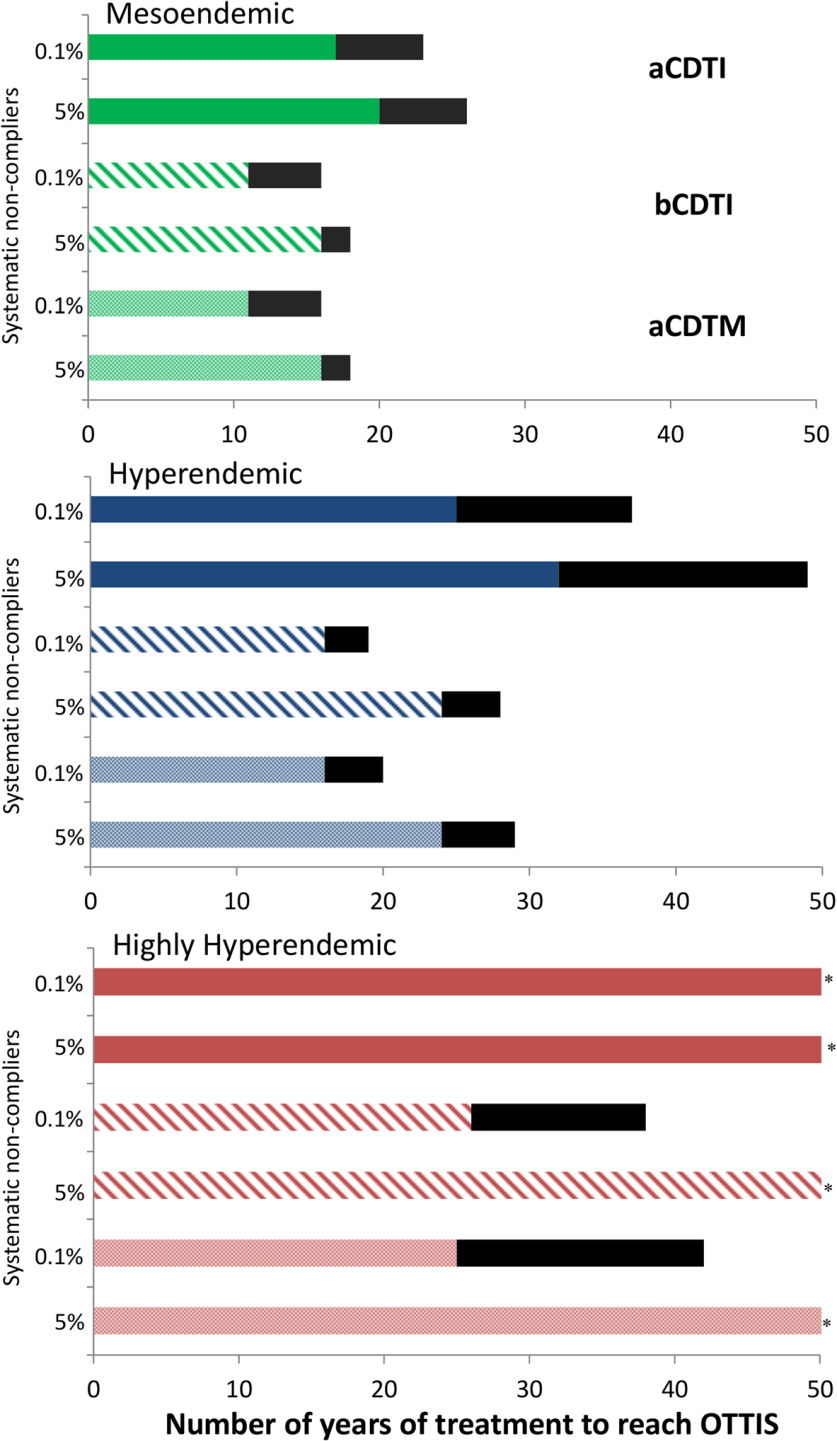
**The effect of coverage and non-compliance on programme duration under strategies of annual and biannual community-directed treatment with ivermectin (aCDTI, bCDTI) and annual community-directed treatment with moxidectin (aCDTM).** Black bars represent the increment in programme duration caused by a decrease in therapeutic coverage from 80% to 60% of the total population. The green, blue and red bars correspond to a pre-control endemicity level of 40%, 60%, and 80% microfilarial prevalence, respectively. aCDTI, bCDTI and aCDTM are indicated by solid, dashed and dotted bars, respectively. Modelling assumptions are as in the legend of Figure 2. *Provisional operational threshold for treatment interruption followed by surveillance (pOTTIS <1.4% microfilarial prevalence) not attained within the 50-year time horizon.

Decreasing therapeutic coverage from 80% to 60% and/or increasing the percentage of systematic non-compliers (those who never take treatment) from 0.1% to 5% markedly increased programme duration for all three strategies (Figure 4, Table 3). The simulated programme durations with aCDTM were notably less sensitive to variation in therapeutic coverage than those with aCDTI. However, similar to aCDTI and bCDTI [12], aCDTM was highly sensitive to assumed proportions of systematic non-compliance (Figure 4, Table 3).

**Table 3:** **Sensitivity of the duration (time to achieve pOTTIS) and relative total cost of annual ivermectin (aCDTI), biannual ivermectin (bCDTI) and annual moxidectin (aCDTM) treatment programmes to the percentage of therapeutic coverage and systematic non-compliance**

**Baseline endemicity level (microfilarial prevalence)**	**Systematic non-compliance**	**80% overall therapeutic coverage**			**60% overall therapeutic coverage**		
		**Projected duration, in years, of treatment programme (relative cost, in percent)**			**Projected duration, in years, of treatment programme (relative cost, in percent)**		
		**aCDTI**	**bCDTI (ǂ)**	**aCDTM (ǂ,†)**	**aCDTI**	**bCDTI (ǂ)**	**aCDTM (ǂ,†)**
Mesoendemic (40%)	0.1%	17	11 (113%)	11 (71%, 63%)	23	14 (108%)	15 (75%, 70%)
	2.0%	18	13 (124%)	13 (78%, 63%)	24	15 (133%)	16 (75%, 66%)
	5.0%	20	16 (136%)	16 (85%, 63%)	26	18 (124%)	19 (80%, 65%)
Hyperendemic (60%)	0.1%	25	16 (116%)	17 (76%, 66%)	37	19 (104%)	21 (70%, 67%)
	2.0%	28	19 (123%)	20 (80%, 65%)	42	22 (108%)	24 (72%, 66%)
	5.0%	32	24 (133%)	24 (83%, 63%)	49	28 (119%)	29 (76%, 64%)
Highly hyperendemic (80%)	0.1%	NA	26 (112%)	26 (70%, 63%)	NA	38 (140%)	46 (96%, 69%)
	2.0%	NA	40 (144%)	43 (80%, 65%)	NA	NA (160%)	NA (100%, 63%)
	5.0%	NA	NA (160%)	NA (100%, 63%)	NA	NA (160%)	NA (100%, 63%)

^ǂ^Percentage cost relative to aCDTI. ^†^Percentage cost relative to bCDTI. NA: Operational thresholds for treatment interruption not attained within the 50-year time horizon (and percentage of costs calculated based on costs of 50 years of treatment). Modelling assumptions are as in the legend of Table 2.

### Impact of anti-macrofilarial action

The data from the single dose Phase II trial do not permit drawing conclusions on the relative effects of moxidectin and ivermectin on adult worm viability or permanent reproductive capacity [17]. There are no data on the potential cumulative anti-macrofilarial activity of repeated annual doses of moxidectin, which is also uncertain for ivermectin [19,21]. The projected programme durations with aCDTM were substantially less sensitive to the assumed anti-macrofilarial action (the per dose cumulative reduction in microfilarial production by female adult worms) than aCDTI or bCDTI within the 1% to 30% range investigated. The difference in programme durations between aCDTM and CDTI (aCDTI or bCDTI) was highest when assuming a very low, 1% anti-macrofilarial action (Table 2, Additional file 1: Table S4). Increasing the assumed anti-macrofilarial action to 30%, markedly reduced the projected programme durations with aCDTI and, to a lesser extent bCDTI, while those with aCDTM were hardly affected. Under all assumptions about the anti-macrofilarial action, the projected programme duration with aCDTM was always clearly shorter than that with aCDTI. With a 30% (and at times 7%) anti-macrofilarial action, bCDTI programmes were one year shorter than aCDTM programmes, but at a notably higher total cost (Table 2, Additional file 1: Table S4).

### Impact of timing of aCDTI and aCDTM in areas with seasonal transmission

**Figure 5:**
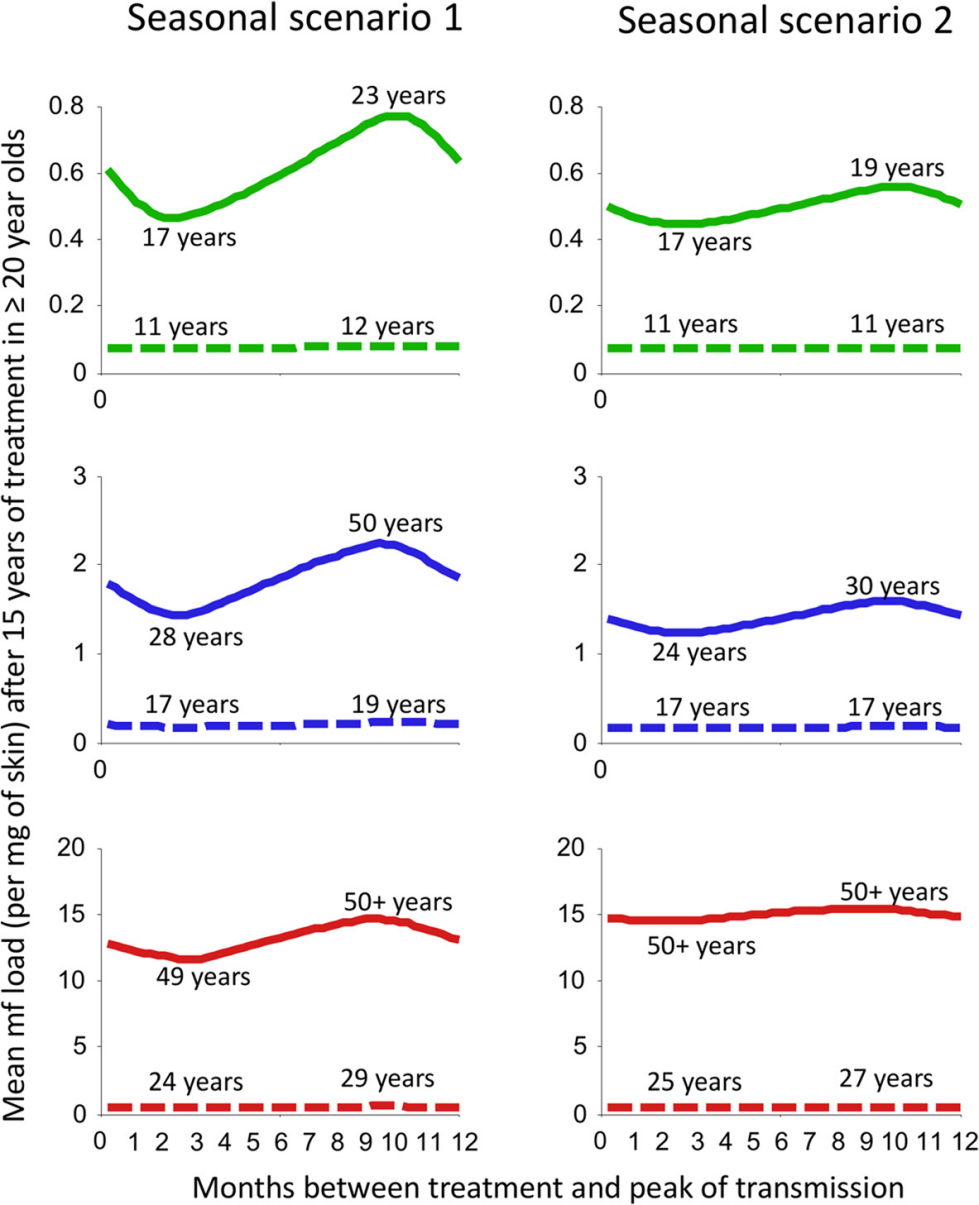
**The effect of the timing of treatment rounds relative to seasonal peaks in transmission by blackfly vectors on programme duration under strategies of annual community-directed treatment with ivermectin (aCDTI) and annual community-directed treatment with moxidectin (aCDTM).** The time between treatment and the peak of transmission was varied. The *x*-axis represents the number of months between treatment at time 0 and the peak of transmission. Scenario 1: transmission only takes place during a five month long rainy season. Scenario 2: high levels of transmission during a five month long rainy season but continuing at a low level during the rest of the year (Table 1 and Additional file 1: Figure S1). The green, blue and red lines correspond to, respectively, a pre-control endemicity level of 40%, 60%, and 80% microfilarial prevalence. The years are those needed to reach the pOTTIS (<1.4% microfilarial prevalence). The lines show the mean microfilarial load per mg of skin after 15 years of treatment in those aged ≥ 20 years. Solid and dashed lines indicate aCDTI and aCDTM respectively. Modelling assumptions are as in the legend of Figure 2.

The timing of aCDTI relative to seasonal transmission peaks had a striking effect on programme duration (Figure 5). The higher the initial endemicity and the more extreme the pattern of seasonal transmission (Figure 5 and Additional file 1: Figure S1), the greater the importance of CDTI timing to ensure maximum reduction in skin microfilarial loads during the peak transmission period. In contrast, timing of aCDTM had little effect on programme duration because of the sustained, year-long suppression of microfilaridermia (Figure 1B).

### In-country costs

Provided moxidectin is donated to endemic countries, the shorter programme durations with aCDTM would lead to substantial in-country cost savings compared to aCDTI, even when aCDTM is assumed to be 10% more costly to deliver than aCDTI (Additional file 1: Table S5). Given that bCDTI costs around 60% more per year than aCDTI [15], the similar programme durations of bCDTI and aCDTM make aCDTM even more cost effective. Cost savings of aCDTM were considerable compared with bCDTI even under scenarios of maximum bCDTI efficacy (assuming a 30% per dose (cumulative) anti-macrofilarial action). Cost savings with aCDTM decreased with increasing discount rates, but costs of aCDTM were at least 19% lower than those of aCDTI and up to 39% lower than those of bCDTI even with a discount rate as high as 6% [23] (Additional file 1: Table S6).

## Discussion

### Programme duration and cost savings

Model outputs indicate that annual moxidectin distribution (aCDTM) is similarly effective for reaching the provisional thresholds for interrupting treatment (pOTTIS) as increasing the frequency of ivermectin distribution from once (aCDTI) to twice per year (bCDTI). This is attributed to the fact that moxidectin reduces skin microfilarial loads faster, more pronouncedly, and for longer than ivermectin (Figure 1), effectively halting transmission between consecutive yearly treatments, akin to a drug that sterilizes the vast majority of adult worms for around one year. This explains why simulated programme durations with aCDTM were not as sensitive as those with aCDTI or bCDTI to the assumed magnitude of anti-macrofilarial action (the per dose reduction in microfilarial production of female adult worms) (Table 2, Additional file 1: Table S4). The modelled anti-macrofilarial efficacy (1%, 7% or 30% per dose cumulative reduction in microfilarial production) with bCDTI accumulates twice as fast as with aCDTI or aCDTM. Despite this, even at an assumed 30% per dose anti-macrofilarial effect, programme durations with bCTDI were only 1 year shorter than those employing aCDTM, due to moxidectin’s pronounced effect on the reproductive activity of the adult worms as modelled in order to reproduce the very late and slow repopulation of skin by microfilariae observed (Figure 1B).

When modelling perennial transmission, the model assumes that bCDTI is given precisely every 6 months, which does not always occur in practice. Delays in treatment distribution will likely increase projected programme durations as they would permit more transmission within the year than the model assumed. This is particularly pertinent to bCDTI, as this would reduce its benefit relative to aCDTI.

The aCDTM strategy was projected to generate marked in-country cost savings (not including the cost of the drug, assumed to be donated) compared to both aCDTI and bCDTI under all assumptions on discount rates, even when each aCDTM round was set to cost 10% more than aCDTI. The largest cost savings occurred when aCDTM was compared with bCDTI, which costs approximately 60% more per year than aCDTI [15].

Both bCDTI and aCDTM substantially reduced the heterogeneity in programme durations for areas with different baseline endemicities. This has high programmatic value because treatment should only be stopped when there is little or no risk of parasite re-introduction from neighbouring areas with continuing transmission. Therefore, aCDTM could be a cheaper alternative to bCDTI where progress towards elimination is lagging, reducing the potential sources of infection in adjoining areas where good progress has been made and thereby protecting the economic and public health investments made.

### Coverage and compliance

While clinical trials of moxidectin have been conducted to date only in participants aged ≥ 12 years [17] (Clinicaltrials.gov/ct2/show/NCT00790998), a paediatric study is part of the moxidectin clinical development plan [17]. Therefore, our modelling assumed that the age groups eligible for ivermectin and moxidectin are identical (≥5 years). Demonstration of the safety of moxidectin in children 5 to 11 years will be crucial to ensure aCDTM has the projected impact.

The longer lasting effects of moxidectin result in aCDTM being substantially less sensitive than aCDTI to the level of therapeutic coverage; aCDTM could thus have particular value where coverage is relatively low due to scarce resources or logistical difficulties; circumstances which would also impede bCDTI. All treatment strategies were acutely and deleteriously affected by increasing levels of systematic non-compliance, highlighting the importance of reducing systematic non-compliance regardless of the treatment strategy. Cost savings generated by aCDTM could be partly invested in social mobilization and other activities that increase compliance. Such initiatives will become more important in the advanced stages of control as decreasing morbidity reduces individuals’ motivation to take treatment.

In highly hyperendemic areas with low coverage and/or high levels of systematic non-compliance, neither aCDTM nor bCDTI was sufficient to reach the pOTTIS within meaningful timeframes. This highlights the importance of alternative and/or complementary strategies including novel macrofilaricidal therapies [24-27]—provided the necessary compliance is achieved—and vector control [28] and, in the longer term, a possible onchocerciasis vaccine [29].

### Timing of treatment

The duration of aCDTI programmes was highly sensitive to the timing of treatment relative to seasonal transmission patterns (Figure 5). This should be considered when evaluating the best strategies for reaching elimination and when smaller than expected reductions in prevalence of infection are observed. Furthermore, in areas with highly seasonal transmission, investment into implementing bCDTI may not significantly reduce programme duration and it could be more cost-effective to invest resources into optimal timing and treatment coverage of aCDTI. This analysis shows the need for further research into the optimum timing of CDTI for all types of seasonal transmission patterns in Africa. This is particularly relevant for decisions on investing additional resources into bCDTI. These investigations should include the impact and cost-effectiveness of increasing overall treatment coverage across the year by treating in the second yearly treatment round individuals not treated in the first round.

The effectiveness of aCDTM was substantially less sensitive to the transmission pattern and thus less vulnerable to factors which affect the actual versus planned timing of treatment (such as drug availability, synergistic resource use in NTD programmes, access to communities, and local community decisions). This is because of moxidectin’s high and prolonged efficacy, which results in almost full, year-long suppression of microfilaridermia (Figure 1B). The aCDTM strategy would also be valuable where political instability or even conflicts make it difficult to guarantee regular and optimal timing of aCDTI or bCDTI.

### Limitations

Many of the methodological limitations of this analysis have been previously discussed [12]; including the fact that the model is currently parameterized for savannah areas of Africa (and not forest settings). Furthermore, the model does not account for any “spill over” infection between neighbouring onchocerciasis foci and therefore underestimates the value in reducing heterogeneity in programme duration among areas with different baseline endemicities and/or control programme performance.

The modelling of in-country costs assumes that moxidectin would be donated to endemic countries and distributed with no or only a 10% increase in costs relative to distribution of ivermectin. WHO has concluded an agreement with the Australian not-for-profit organization Medicines Development for Global Health (MDL, http://www.medicinesdevelopment.com/) to transfer all moxidectin-related data to MDL. MDL intends to register moxidectin, initially for onchocerciasis, and ensure manufacturing. The validity of this assumption is thus expected to become clearer over the next couple of years.

The fit of our model to the skin microfilarial density data from the Phase II trial (Figure 1) requires validation against the dataset from the Phase III trial [30]. That dataset is not as appropriate as the Phase II trial data set for fitting the microfilarial temporal dynamics after moxidectin treatment because it comprises fewer post-treatment time points. However, it includes skin microfilarial densities from around 25 times as many people as the Phase II trial. The ongoing analysis of the Phase III study data [30] suggests that the curve derived from the Phase II trial (Figure 1) provides a good fit to the Phase III data on skin microfilarial densities.

EPIONCHO is a deterministic model where all events occur in a pre-specified way depending on the parameter values and initial conditions of the model. It therefore does not account for the influence of random events and, in the context of elimination, the phenomenon of stochastic fade-out [31] (chance elimination of the parasite at low population densities). Consequently, EPIONCHO cannot be used to investigate formally the probability of reaching elimination. Furthermore, it is parameterized for the savannah species of the *S. damnosum s.l.* vector complex (*S. damnosum s. str*. and *S. sirbanum*) [18,32], and the influence of different combinations of vectors on the impact of control requires further investigation. Finally, it is important to consider that most models of helminth transmission dynamics (including EPIONCHO) are parameterized with data collected before commencement of control and formal validation against longitudinal data from ongoing interventions is scant. It is possible that the relationships between infection and transmission could be influenced by the treatment [33]. Therefore, any model-derived predictions of the long-term impact of treatment have a degree of uncertainty.

### Other considerations relating to the use of moxidectin

Moxidectin exerts a more potent initial microfilaricidal effect than ivermectin [17]. Consequently, it is likely that moxidectin, like ivermectin, will be contraindicated in patients with heavy *Loa loa* co-infections because of the risk of severe and/or serious adverse events associated with rapid microfilaricidal activity against *L. loa* microfilariae [24,34]. This highlights the need for drugs without microfilaricidal activity [24-26] and complementary vector control strategies for *L. loa* co-endemic areas. Alternatively, moxidectin could be considered in these areas within the ‘test for loiasis and do not treat’ strategy now being explored for ivermectin (possibly also including a test for onchocerciasis and alternative treatment), to identify and exclude individuals at risk of severe and/serious adverse reactions to treatment. In that case, the applicability of risk thresholds determined for ivermectin needs to be carefully considered.

The pOTTIS are provisional operational thresholds for stopping treatment followed by surveillance based on the experience with vector control in the OCP, aCDTI and bCDTI in Mali and Senegal and projections from (other) transmission models [7,8,13]. They include the residual level of patent infection across the whole age range assessed via skin microfilarial prevalence. The longer lasting effect of moxidectin on microfilarial production by female worms, if shown not to be associated with an irreversible effect after multiple doses, needs to be considered in the choice of time for the assessment post treatment. This is not the case if antibody prevalence in 1–5 year olds, born after the presumed interruption of transmission, is used as one of the criteria for stopping treatment as in the 2001 WHO guidelines for certification of onchocerciasis elimination, developed in view of elimination in the Americas [35] and currently under review.

Furthermore, it is important to reiterate that the current pOTTIS are provisional and not necessarily equivalent to the true transmission breakpoint for elimination in all settings. In particular, in areas with very high pre-control endemicity levels, with high vector biting rates, the breakpoint may be lower than the pOTTIS [36]. However, this is unlikely to influence the relative benefit of moxidectin compared to ivermectin regarding reduced programme duration, as seen by the consistency of the relative benefit over the different values of the pOTTIS applied in the sensitivity analysis (Figures 2 and 3).

In the context of integrated NTD control, there is renewed interest in the broader antiparasitic properties of ivermectin and integration of strategies for controlling onchocerciasis and other helminthiases, particularly lymphatic filariasis and soil-transmitted helminthiasis [16]. Scabies is another infection treatable with ivermectin for which evaluation of moxidectin has been proposed [37]. It will thus be important to establish whether moxidectin has similar effects to those of ivermectin on a range of human parasites—as data from veterinary use suggest [16] —and, therefore, whether it has strong potential for integrated control of helminth and other infections. This is particularly important now that APOC may become a new regional entity, the Programme for the Elimination of Neglected Diseases in Africa (PENDA), with a wider mandate to tackle all the five preventive chemotherapy diseases, including lymphatic filariasis and onchocerciasis elimination [38,39].

## Conclusions

Annual CDTM could result in achieving the proposed thresholds for stopping treatment in Africa within timeframes comparable to those achievable with biannual CDTI, but at a substantially lower cost to countries (provided moxidectin is donated). Moreover, the effectiveness of annual CDTM is essentially impervious to seasonal peaks in transmission, whereas suboptimal timing of annual CDTI can significantly reduce its effectiveness. Moxidectin, therefore, represents a potentially superior alternative drug for onchocerciasis control and elimination.

## Additional file

Additional file 1:
**Supporting Information.** **Text S1.** Seasonality of transmission. **Figure S1.** Investigated scenarios of seasonal transmission, illustrated for a pre-control endemicity of 40% microfilarial prevalence. **Figure S2.** Schematic representation of the model and the corresponding differential equations. **Text S2**. Estimating the dynamic effects on skin microfilarial loads induced by treatment with moxidectin. **Table S1.** Parameter definitions and values describing seasonality in onchocerciasis transmission. **Table S2.** Definitions and values of parameters and variables determining microfilarial load dynamics following treatment with moxidectin. **Table S3.** Summary of pre-control epidemiology (perennial transmission), African savannah. **Table S4.** Sensitivity to the magnitude of the assumed anti-macrofilarial action of ivermectin and moxidectin of the additional programme duration and cost of switching from annual community-directed treatment with ivermectin (aCDTI) to biannual CDTI (bCDTI) or annual community directed treatment with moxidectin (aCDTM). **Table S5.** In-country costs to reach provisional operational threshold for treatment interruption followed by surveillance (pOTTIS) of annual community-directed treatment with moxidectin (aCDTM) relative to community-directed treatment with ivermectin (CDTI) for two assumptions on the cost of implementing aCDTM. **Table S6.** Sensitivity to the assumed discount rate of the relative total programme cost of annual community-directed treatment with moxidectin (aCDTM) compared to annual or biannual community-directed treatment with ivermectin (aCDTI, bCDTI).

## Abbreviations

ABR: Annual biting rate
aCDTI: Annual community-directed treatment with ivermectin
aCDTM: Annual community-directed treatment with moxidectin
APOC: African Programme for Onchocerciasis Control
bCDTI: Biannual (6-monthly) community-directed treatment with ivermectin
CDTI: Community-directed treatment with ivermectin
MDL: Medicines Development for Global Health
NGO: Non-governmental organization
NTD: Neglected tropical disease
OCP: Onchocerciasis Control Programme in West Africa
PENDA: Programme for the Elimination of Neglected Diseases in Africa
pOTTIS: Provisional operational thresholds for treatment interruption followed by surveillance
WHO: World Health Organization
