# Supplementary material for: The potential impact of moxidectin on onchocerciasis elimination in Africa: an economic evaluation based on the Phase II clinical trial data
Source: Parasit Vectors. 2015 Mar 19;8:167. doi: 10.1186/s13071-015-0779-4 (PMC4381491; doi:10.1186/s13071-015-0779-4)
Supplement: Additional file 1: — Supporting Information. Text S1. Seasonality of transmission. Figure S1. Investigated scenarios of seasonal transmission, illustrated for a pre-control endemicity of 40% microfilarial prevalence. Figure S2. Schematic representation of the model and the corresponding differential equations. Text S2. Estimating the dynamic effects on skin microfilarial loads induced by treatment with moxidectin. Table S1. Parameter definitions and values describing seasonality in onchocerciasis transmission. Table S2. Definitions and values of parameters and variables determining microfilarial load dynamics following treatment with moxidectin. Table S3. Summary of pre-control epidemiology (perennial transmission), African savannah. Table S4. Sensitivity to the magnitude of the assumed anti-macrofilarial action of ivermectin and moxidectin of the additional programme duration and cost of switching from annual community-directed treatment with ivermectin (aCDTI) to biannual CDTI (bCDTI) or annual community directed treatment with moxidectin (aCDTM). Table S5. In-country costs to reach provisional operational threshold for treatment interruption followed by surveillance (pOTTIS) of annual community-directed treatment with moxidectin (aCDTM) relative to community-directed treatment with ivermectin (CDTI) for two assumptions on the cost of implementing aCDTM. Table S6. Sensitivity to the assumed discount rate of the relative total programme cost of annual community-directed treatment with moxidectin (aCDTM) compared to annual or biannual community-directed treatment with ivermectin (aCDTI, bCDTI). [file 13071_2015_779_MOESM1_ESM.pdf]

Supporting Information

S1. Seasonality of transmission

EpiOncho was modified to permit the instantaneous biting rate (the number of bites received per person per unit time) of blackfly vectors to vary within the year to reflect seasonality in the transmission of onchocerciasis. It is assumed that in the absence of vector control operations, the entomological conditions during the treatment programme remain unchanged. A sinusoidal functional form recently used to model seasonality in malaria transmission [1] was parameterized to reflect two scenarios of seasonal transmission (Figure S1 and Table S1) typical in Africa:

Seasonal transmission scenario 1: An extreme scenario with transmission occurring during a rainy season typically lasting between four to five months each year; based on the entomological situation in foci of Senegal, Mali [2, 3] and Nigeria [4], where elimination has been reported.

Seasonal transmission scenario 2: A longer period of transmission, still peaking in the rainy season but not ceasing completely in the dry season; motivated by the entomological observations conducted in Nigeria and reported in [5].

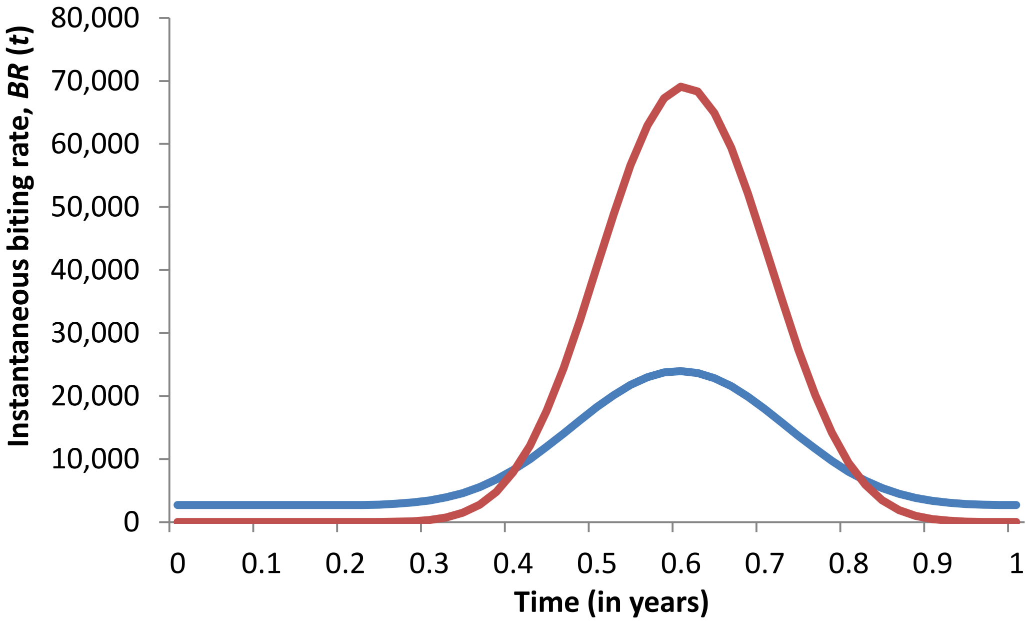

Figure S1: Investigated scenarios of seasonal transmission, illustrated for a pre-control endemicity of 40% microfilarial prevalence. The red and blue lines correspond to, respectively, seasonal transmission scenario 1 and seasonal transmission scenario 2 (see text above). The area under the curve (i.e. the average instantaneous biting rate over the year) corresponds to the annual biting rate or ABR (17,038 bites/person/yr for scenario 1 and 9,148 bites/person/yr for scenario 2 – the difference in the ABR's is due to the (negative) density dependent processes governing the establishment of incoming worms within the human host [6, 7]). The parameter definitions and values are presented in Table S1.

Supplementary Table S1. Parameter definitions and values describing seasonality in onchocerciasis transmission

Symbol	Definition of variables and parameters	Expression, average value and units	Ref.
BR(*t*)	Instantaneous biting rate at time *t*	$X\left(c+(1-c)\left\{\frac{1+\cos[2\pi(t-u)]}{2}\right\}^{\kappa}\right)$	[1]
X	The peak instantaneous biting rate	*40% pre-control mf prevalence*<br>Scenario 1: 69,281<br>Scenario 2: 24,019<br>*60% pre-control mf prevalence*<br>Scenario 1: 223,438<br>Scenario 2: 56,688<br>*80% pre-control mf prevalence*<br>Scenario 1: 489,531<br>Scenario 2: 231,281	This work
c	Determines the minimum instantaneous biting rate (=*c*X*, with $0 \le c \le 1$)	Scenario 1: 0<br>Scenario 2: 0.112	This work
u	The position of the transmission peak in relation to treatment timing	Scenario 1: Varied<br>Scenario 2: Varied	This work
κ	Shape parameter describing the biting pattern (for $\kappa > 0$)	Scenario 1: 4.9<br>Scenario 2: 3.2	This work
ABR	Annual biting rate, no. of bites/person/year	$ABR = \int_{t=0}^{t=1} BR(t)dt$, for $u = 0$	This work
β	Biting rate per fly on humans assuming a human blood index of 0.3 and biting every 3.5 days	31.25 yr^{-1}	[7, 8]
$m(t)$	Vector to host ratio at time (*t*)	$BR(t) / \beta$	

Scenarios are described in section *S.1.Seasonality of transmission.* mf prevalence: microfilarial prevalence.

S.2.Estimating the dynamic effects on skin microfilarial loads induced by treatment with moxidectin

The following differential equations and illustration (Figure S2) describe the rate of change with respect to time of the mean number of non-fertile (*N*) and fertile (*F*) adult female worms per person, and the mean number of microfilariae per mg of skin (*M*), parameters defined in Supporting Table S.2.

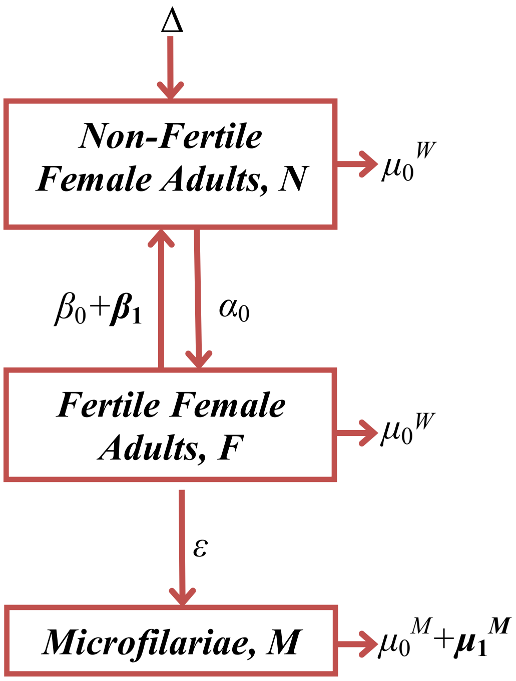

$$\frac{dN}{dt} = \Delta + (\beta_0 + \beta_1(\tau))F - (a_0 + \mu_0^W)N$$

$$\frac{dF}{dt} = a_0 N - (\beta_0 + \beta_1(\tau) + \mu_0^W)F$$

$$\frac{dM}{dt} = \varepsilon F - (\mu_0^M + \mu_1^M(\tau))M$$

Figure S2: Schematic representation of the model and the corresponding differential equations. The parameter definitions and values are presented in Table S2.

The model is identical to that used in a previous meta-analysis to estimate the dynamic effects of ivermectin on microfilarial loads [9]. Here, $\mu_1^M(\tau)$ (the capita death rate of microfilariae following treatment) and $\beta_1(\tau)$ (the treatment-induced per capita rate at which fertile females become non-fertile) were re-estimated by fitting the model to phase II moxidectin clinical trial data [10] on the percentage reduction from pre-treatment in mean microfilarial load at different times after treatment with 8 mg moxidectin (8 days; 1, 2, 3, 6, 12 and 18 months, Figure 1). Both of these rates depend on the time since treatment, τ . It was assumed that a single 8 mg dose of moxidectin has no macrofilaricidal effect (i.e. does not kill the adult worms, i.e. the macrofilariae). The model was fitted to the data by (nonlinear) least-squares regression, following the approach taken in [9], yielding parameter estimates presented in Table S2

Supplementary Table S2. Definitions and values of parameters and variables determining microfilarial load dynamics following treatment with moxidectin

Symbol	Definition of variables and parameters	Expression, average value and units	Ref.
Δ	Rate at which incoming, initially non-reproducing, female worms establish within onchocercal nodules (i.e. the force of infection)	Arbitrary value, yr^{-1}	
μ_0^W	Per capita death rate of adult worms	0.1 yr^{-1}	[6]
α_0	Per capita rate at which untreated non-reproducing female worms become fertile	0.59 yr^{-1}	[9]
β_0	Per capita rate at which untreated fertile female worms become naturally non-fertile	0.33 yr^{-1}	[9]
ε	Rate of production of microfilariae per fertile female worm scaled by the total weight (in milligrams) of microfilariae-bearing skin	1.1538 yr^{-1}	[6, 11]
μ_0^M	Per capita death rate of microfilariae in the absence of treatment	0.8 yr^{-1}	[6]
τ	Time since last treatment	years	–
$\beta_1(\tau)$	Excess per capita rate at which fertile female worms become non-fertile following treatment (embryostatic effect)	$\beta_1 = \beta_1^{MAX} \exp(-\gamma\ \tau)$ yr^{-1}	[9]
β_1^{MAX}	The maximum per capita rate of treatment-induced sterility	Moxidectin: 462 yr^{-1} (*)<br>Ivermectin: 32.4 yr^{-1}	This work<br>[9]
γ	The rate of decay of treatment-induced sterility with time after treatment	Moxidectin: 4.83 yr^{-1}(*)<br>Ivermectin: 19.6 yr^{-1}	This work<br>[9]
$\mu_1^M(\tau)$	Excess per capita death rate of microfilariae following treatment (microfilaricidal effect)	$\mu_1^M = (\tau + \nu)^{-\varpi}$ yr^{-1}	[9]
ν	Constant added to time after treatment to allow for a very large, yet finite, microfilaricidal effect at the point of treatment	Moxidectin: 0.04 (*)<br>Ivermectin: 0.0096	This work<br>[9]
ϖ	Shape parameter for the per capita death rate of microfilariae following treatment	Moxidectin: 1.82 (*)<br>Ivermectin: 1.25	This work<br>[9]

*The values indicated with an asterisk were estimated in this work as described in the legend of Figure S2.

Supplementary Table S3: Summary of pre-control epidemiology (perennial transmission), African savannah

Pre-control endemicity level	Microfilarial prevalence	Annual biting rate[§†]	Annual transmission potential[¶†]	Mean intensity[*] (mf per mg)	Mean intensity[*] in those aged ≥ 20 (mf per mg)
Mesoendemic	40%	7,305	88	11.2	18.7
Hyperendemic	60%	15,472	373	23.9	40.0
Highly hyperendemic	80%	85,800	4,290	58.9	98.0

[§] Annual biting rate (ABR): the average number of *Simulium damnosum s.s./ S. sirbanum* bites to which a person is exposed during a whole year.

[¶] Annual transmission potential (ATP): the average number of infective larvae (L3) of *Onchocerca volvulus* potentially received during a whole year by a person exposed to the annual biting rate.

[†] Both the ABR and ATP are for a proportion of vector blood meals of human origin equal to 0.3 [6].

[*] Arithmetic mean microfilarial load per mg of skin; note that this is different to the community microfilarial load (CMFL), which is the geometric mean microfilarial load per skin snip in those aged 20 years and above) [12].

Supplementary Table S4: Sensitivity to the magnitude of the assumed anti-macrofilarial action of ivermectin and moxidectin of the additional programme duration and cost of switching from annual community-directed treatment with ivermectin (aCDTI) to biannual CDTI (bCDTI) or annual community directed treatment with moxidectin (aCDTM)

Schedule of ivermectin treatment strategy and initial level of onchocerciasis endemicity		1% cumulative reduction in microfilarial production by female adult worms per dose			7% cumulative reduction in microfilarial production by female adult worms per dose			30% cumulative reduction in microfilarial production by female adult worms per dose		
		Projected duration, in years, of treatment programme (relative cost, in percent)			Projected duration, in years, of treatment programme (relative cost, in percent)			Projected duration, in years, of treatment programme (relative cost, in percent)		
		aCDTI	bCDTI (‡)	aCDTM(‡,†)	aCDTI	bCDTI(‡)	aCDTM(‡,†)	aCDTI	bCDTI(‡)	aCDTM(‡,†)
**Switching to bCDTI or aCDTM treatment at different levels of prevalence in an ongoing aCDTI programme**										
30% microfilarial prevalence	Mesoendemic	20	13(115%)	11(63%,55%)	16	10(109%)	10(68%,62%)	11	8(122%)	9(84%,69%)
	Hyperendemic	32	19(113%)	17(65%,58%)	24	16(119%)	16(74%,62%)	16	14(144%)	14(90%,63%)
	Highly hyperendemic	NA	31(125%)	24(66%,53%)	NA	21(97%)	22(63%,65%)	34	19(109%)	20(71%,65%)
20% microfilarial prevalence	Mesoendemic	20	13(115%)	11(63%,55%)	16	10(109%)	10(68%,62%)	11	8(122%)	9(84%,69%)
	Hyperendemic	29	17(110%)	15(63%,57%)	21	13(111%)	13(69%,62%)	15	13(143%)	13(89%,63%)
	Highly hyperendemic	NA	26(112%)	20(58%,52%)	NA	18(87%)	19(56%,64%)	32	18(109%)	19(71%,65%)
15% microfilarial prevalence	Mesoendemic	20	13(115%)	11(63%,55%)	16	10(109%)	10(68%,62%)	11	8(122%)	9(84%,69%)
	Hyperendemic	25	14(104%)	12(58%,56%)	19	11(104%)	12(70%,67%)	13	11(139%)	12(94%,68%)
	Highly hyperendemic	NA	22(100%)	17(52%,52%)	NA	15(75%)	17(52%,69%)	30	17(108%)	18(71%,66%)

‡ Percentage cost relative to aCDTI. † Percentage cost relative to bCDTI. NA: Operational thresholds for treatment interruption not attained within the 50-year time horizon (and percentage of costs calculated based on costs of 50 years of treatment). CDTM: community-directed treatment moxidectin, CDTI: community-directed treatment with ivermectin. This analysis was performed with a 50-year time horizon, discount rate of 3% applied to the costs, therapeutic coverage of 80%, 0.1% of systematic non-compliers, perennial transmission and pOTTIS <1.4% microfilarial prevalence. Costs do not include the value of the (donated) drugs.

Supplementary Table S5: In-country costs to reach provisional operational threshold for treatment interruption followed by surveillance (pOTTIS) of annual community-directed treatment with moxidectin (aCDTM) relative to community-directed treatment with ivermectin (CDTI) for two assumptions on the cost of implementing aCDTM.

Schedule of treatment strategy and initial level of onchocerciasis endemicity		Cost (per year) of aCDTM relative to aCDTI	
		Same	10% higher
**Annual moxidectin treatment implemented from start of the programme**		**Percentage of the total cost of aCDTM relative to aCDTI and bCDTI respectively**	
	Mesoendemic	71%, 63%	78%, 69%
	Hyperendemic	76%, 65%	84%, 72%
	Highly hyperendemic	70%, 63%	77%, 69%
**Switching to moxidectin treatment at different levels of microfilarial prevalence during an ongoing annual treatment programme**		**Additional total costs* of aCDTM relative to continuing aCDTI, and switching to bCDTI**	
30% microfilarial prevalence	Mesoendemic	68%, 60%	75%, 66%
	Hyperendemic	74%, 62%	81%, 68%
	Highly hyperendemic	63%, 65%	69%, 71%
20% microfilarial prevalence	Mesoendemic	68%, 62%	75%, 69%
	Hyperendemic	69%, 62%	76%, 68%
	Highly hyperendemic	56%, 64%	62%, 71%
15% microfilarial prevalence	Mesoendemic	68%, 62%	75%, 69%
	Hyperendemic	70%, 67%	77%, 74%
	Highly hyperendemic	52%, 69%	57%, 76%

aCDTM: annual community-directed treatment moxidectin * The ratio of additional costs is considered from the point of switching from annual to biannual treatment (as opposed to from the start of control). When switching from annual to biannual treatment, infection (microfilarial) prevalence was assumed to be measured just before the next round of treatment distribution. The analysis was performed with a 50-year time horizon, discount rate of 3% applied to the costs, therapeutic coverage of 80%, 0.1% of systematic non-compliers, perennial transmission, and 7% cumulative reduction in microfilarial production by female adult worms per treatment dose. pOTTIS < 1.4% microfilarial prevalence. Costs do not include the value of the (donated) drugs.

Supplementary Table S6: Sensitivity to the assumed discount rate of the relative total programme cost of annual community-directed treatment with moxidectin (aCDTM) compared to annual or biannual community-directed treatment with ivermectin (aCDTI, bCDTI)

**Treatment strategy and initial level of onchocerciasis endemicity**		**Total costs of aCDTM in % of costs of aCDTI, and bCDTI**		
		**Discount rate**		
**aCDTM implemented from start of the programme**		0%	3%	6%
	Mesoendemic	65%, 63%	71%, 63%	76%, 62%
	Hyperendemic	68%, 66%	76%, 66%	83%, 65%
	Highly hyperendemic	52%, 63%	70%, 63%	84%, 63%
**Switching to aCDTM at different levels of microfilarial prevalence in an ongoing annual CDTI programme**		**Additional total costs* of aCDTM relative to continuing aCDTI, and switching to bCDTI**		
30% microfilarial prevalence	Mesoendemic	63%, 61%	68%, 60%	74%, 61%
	Hyperendemic	67%, 63%	74%, 62%	81%, 62%
	Highly hyperendemic	44%, 66%	63%, 65%	78%, 64%
20% microfilarial prevalence	Mesoendemic	63%, 63%	68%, 62%	74%, 63%
	Hyperendemic	62%, 63%	69%, 62%	76%, 62%
	Highly hyperendemic	38%, 66%	56%, 64%	73%, 65%
15% microfilarial prevalence	Mesoendemic	63%, 63%	68%, 62%	74%, 63%
	Hyperendemic	63%, 68%	70%, 67%	76%, 67%
	Highly hyperendemic	34%, 71%	52%, 69%	68%, 67%

aCDTM: annual community-directed treatment moxidectin, aCDTI: annual community-directed treatment with ivermectin, bCDTI: biannual community-directed treatment with ivermectin. * The ratio of additional costs is considered from the point of switching from annual to biannual treatment (as opposed to from the start of control). Modelling assumptions are as in the legend of Table S5.

References

1. Griffin JT, Hollingsworth TD, Okell LC, Churcher TS, White M, Hinsley W, Bousema T, Drakeley CJ, Ferguson NM, Basáñez M-G *et al*: **Reducing *Plasmodium falciparum* malaria transmission in Africa: A model-based evaluation of intervention strategies**. *PLoS Med* 2010, **7**(8):e1000324.
2. Diawara L, Traoré MO, Badji A, Bissan Y, Doumbia K, Goita SF, Konaté L, Mounkoro K, Sarr MD, Seck AF *et al*: **Feasibility of onchocerciasis elimination with ivermectin treatment in endemic foci in Africa: first evidence from studies in Mali and Senegal**. *PLoS Negl Trop Dis* 2009, **3**(7):e497.
3. Traoré MO, Sarr MD, Badji A, Bissan Y, Diawara L, Doumbia K, Goita SF, Konate L, Mounkoro K, Seck AF *et al*: **Proof-of-principle of onchocerciasis elimination with ivermectin treatment in endemic foci in Africa: final results of a study in Mali and Senegal**. *PLoS Negl Trop Dis* 2012, **6**(9):e1825.
4. Tekle A, Elhassan E, Isiyaku S, Amazigo U, Bush S, Noma M, Cousens S, Abiose A, Remme JHF: **Impact of long-term treatment of onchocerciasis with ivermectin in Kaduna State, Nigeria: first evidence of the potential for elimination in the operational area of the African Programme for Onchocerciasis Control**. *Parasit Vectors* 2012, **5**(1):28.
5. Opara KN, Fagbemi OB, Ekwe A, Okenu DM: **Status of forest onchocerciasis in the Lower Cross River basin, Nigeria: entomologic profile after five years of ivermectin intervention**. *Am J Trop Med Hyg* 2005, **73**(2):371-376.
6. Basáñez MG, Boussinesq M: **Population biology of human onchocerciasis**. *Philos Trans R Soc Lond B Biol Sci* 1999, **354**(1384):809-826.
7. Filipe JAN, Boussinesq M, Renz A, Collins RC, Vivas-Martinez S, Grillet ME, Little MP, Basáñez MG: **Human infection patterns and heterogeneous exposure in river blindness**. *Proc Natl Acad Sci U S A* 2005, **102**(42):15265-15270.
8. Basáñez MG, Collins RC, Porter CH, Little MP, Brandling-Bennett D: **Transmission intensity and the patterns of *Onchocerca volvulus* infection in human communities**. *Am J Trop Med Hyg* 2002, **67**(6):669-679.
9. Basáñez MG, Pion SDS, Boakes E, Filipe JAN, Churcher TS, Boussinesq M: **Effect of single-dose ivermectin on *Onchocerca volvulus*: a systematic review and meta-analysis**. *Lancet Infect Dis* 2008, **8**(5):310-322.
10. Awadzi K, Opoku NO, Attah SK, Lazdins-Helds J, Kuesel AC: **A randomized, single-ascending-dose, ivermectin-controlled, double-blind study of moxidectin in Onchocerca volvulus infection**. *PLoS Negl Trop Dis* 2014, **8**(6):e2953.
11. Duke BOL: **The population dynamics of *Onchocerca volvulus* in the human host**. *Trop Med Parasitol* 1993, **44**(2):61-68.
12. Remme JHF, Ba O, Dadzie KY, Karam M: **A force-of-infection model for onchocerciasis and its applications in the epidemiological evaluation of the Onchocerciasis Control Programme in the Volta River basin area**. *Bull World Health Organ* 1986, **64**(5):667-681.
